# Early Knee Osteoarthritis Detection by Multi-Component T_2_ Mapping

**DOI:** 10.3390/bioengineering13030348

**Published:** 2026-03-17

**Authors:** Hector L. de Moura, Anmol Monga, Dilbag Singh, Marcelo V. W. Zibetti, Jonathan Samuels, Ravinder R. Regatte

**Affiliations:** 1 Department of Radiology, New York University Grossman School of Medicine, New York, NY 10016, USA; anmol.monga@nyulangone.org (A.M.); dilbag.singh@nyulangone.org (D.S.); marcelo.wustzibetti@nyulangone.org (M.V.W.Z.); 2 Department of Medicine, New York University Grossman School of Medicine, New York, NY 10016, USA; jonathan.samuels@nyulangone.org

**Keywords:** knee cartilage, osteoarthritis, quantitative MRI, linear discriminative analysis

## Abstract

This study investigates whether multi-component T_2_ mapping, using bi-exponential (BE) and stretched-exponential (SE) models, enhances the early detection of knee osteoarthritis (OA) compared with the conventional mono-exponential (ME) approach. T_2_ relaxation maps were derived from 26 patients with early-stage OA and 26 healthy controls. To minimize the influence of age-related cartilage changes, all model-derived parameters were adjusted for age prior to analysis. Quantitative T_2_ parameters were extracted from six anatomically defined cartilage sub-regions to capture spatially heterogeneous tissue alterations characteristic of early OA. These parameters were then integrated using linear discriminant analysis to assess combined diagnostic performance. Global whole-cartilage analyses demonstrated limited discriminatory power across all models, with area under the receiver operating characteristic curve (AUC) values not exceeding 0.65, indicating that diffuse averaging obscures subtle, localized degeneration. In contrast, sub-regional analysis improved classification accuracy, highlighting the importance of regional assessment in early disease. Among the evaluated models, the BE-T_2_ model showed the highest performance, achieving an AUC of 0.68, and marginally outperforming both the SE model (AUC = 0.60) and the ME model (AUC = 0.51). These findings suggest that multi-component T_2_ mapping, particularly when applied at a sub-regional level, may offer improved sensitivity to early cartilage compositional changes. Overall, this approach shows strong potential as a noninvasive imaging biomarker for the early detection of knee OA.

## 1. Introduction

Osteoarthritis (OA) is a common cause of chronic disability [1,2] and presents a grave socioeconomic burden worldwide [3,4]. During the early stages, the disease causes biochemical and compositional changes in the extracellular matrix, such as proteoglycan depletion and alterations in the collagen network [5]. These changes happen before any structural damage can be detected by radiography or standard magnetic resonance imaging (MRI) [6]. Identifying the disease in the pre-radiographic stage can help guide decisions such as lifestyle changes to slow down disease progression [7]. Additionally, early detection can improve cohort selection for disease-modifying drug studies [8]. Initiatives such as the Foundation for the National Institutes of Health (FNIH) OA Biomarkers Consortium [9] have championed the search for biomarkers that can enrich clinical trials.

Quantitative MRI methods, such as the spin–spin relaxation time (T_2_) and the spin–lattice relaxation time in the rotating frame (T_1ρ_) mapping, offer non-invasive methods to probe the biochemical composition of articular cartilage [7,10]. T_2_ relaxation is primarily affected by water content and organization/orientation of the collagen fibril network, while T_1ρ_ relaxation is also sensitive to proteoglycans [10]. Both T_2_ and T_1ρ_, expressed as the relaxation constant in a mono-exponential (ME) model, showed the capacity to differentiate healthy controls from early OA patients, classified as Kellgren–Lawrence scale 1 and 2 (KL1-2), and are also correlated with OA progression [10]. Still, their translation to clinical routine has been hindered by a lack of standardization and evidence of added value [11]. Detecting early OA remains challenging because compositional changes are subtle and heterogeneous [12], which limits the discriminative performance of any single quantitative MRI metric.

While multi-component models may have theoretical advantages, their clinical utility needs further validation. Previous technical studies have established the feasibility and repeatability of acquiring bi-exponential (BE) and stretched-exponential (SE) T_2_ and T_1ρ_ in vivo [13,14,15] and optimized acquisition parameters to reduce scan times [16]. A recent study [17] evaluated the diagnostic performance of these models for T_1ρ_ mapping, using the Area Under the Curve (AUC) of a classification model that combines the parameters measured at each cartilage compartment. The results showed that multi-regional features are more sensitive to changes in the cartilage.

In this study, T_2_ measurements were analyzed using BE and SE models and compared with the standard ME approach. Guided by earlier findings, we hypothesized that multi-exponential models may offer improved sensitivity compared with the ME model for identifying early osteoarthritic changes. Given the subtle nature of early OA and the modest sample size, this study was designed as a proof-of-concept evaluation of whether regional multi-exponential T2 features offer added sensitivity beyond the conventional ME approach.

## 2. Materials and Methods

This study was approved by the Institutional Review Board (IRB) and conducted in accordance with the Health Insurance Portability and Accountability Act (HIPAA). Written informed consent was obtained from all participants prior to MRI acquisition.

### 2.1. Study Population

This case–control study included 26 healthy subjects (HS; 10 females; mean age 51.5 ± 8.4 years; mean body mass index [BMI] 27.3; no knee pain and Kellgren–Lawrence [KL] grade 0 on radiographs) and 26 patients with early knee osteoarthritis (OA; 19 females; mean age 61.8 ± 7.6 years; mean BMI 27.8; KL grades 1–2). Participant demographics are summarized in Table 1.

Groups were not fully age-matched, which represents a limitation of the dataset. However, because this work is intended as a proof-of-concept evaluation of regional T2 sensitivity, rather than a matched epidemiological comparison, strict age-matching was not required for the study’s primary objective.

All OA patients underwent standardized, weight-bearing, fixed-flexion posteroanterior knee radiography using a SynaFlexer X-ray Positioning Frame (Synarc, San Francisco, CA, USA), as described in [18]. Inclusion criteria for healthy volunteers were age in the range of 40–75 years, either sex, no knee pain or clinical symptoms of OA, i.e., Kellgren–Lawrence (KL) grade of 0, and no abnormal findings on clinical MR protocol, such as meniscal or ligamental tears, assessed by musculoskeletal radiologists in the Division of Radiology at NYU Langone Health. For early OA patients, the inclusion criteria were an age range of 40–75 years, either sex, frequent knee pain on most days of the month for the past year, a KL grade of 1 or 2, and a KL grade of ≤1 in the contralateral knee.

### 2.2. In Vivo Imaging

All subjects were scanned at a 3T scanner (MAGNETOM Prisma, Siemens Healthineers AG, Forchheim, Germany) with a vendor-provided 1-Tx/15-Rx knee coil (QED, OH).

T_2_ images were acquired using a 3D Turbo FLASH [13,19] sequence with matrix size 256 × 128 × 64, interpolated to 256 × 256 × 64, and with a voxel size of 0.8 mm × 0.8 mm × 2 mm. The T_2_ preparation used hard 90° pulses for tip-down and tip-up, along with two hard 180° pulses for refocusing positioned at a quarter and three-quarters of the total preparation time (pTE).

The pTE schedule was optimized using the Cramer–Rao Lower Bound method (CRLB) [16,19] using a range of pTEs between 0.1 and 55 ms with 0.1 ms increments. This optimization resulted in 7 weighted images with TSLs of 0, 4.3, 9, 33, 33, 55, and 55 ms. The repetition of 33 and 55 ms preparations boosts the SNR of the longer preparations, as shown in a previous study. The scan time for each image was 3 min and 12 s, totaling 22 min and 24 s for the 7 pTEs.

### 2.3. Image Processing and Signal Modelling

T_2_ images were reconstructed using a soft-sensitivity encoding (SENSE) approach with the coil-sensitivity maps derived from the eigen decomposition of the calibration data. To preserve phase coherence, only the main component of the decomposition was used for reconstruction. All images from the same acquisition were rigidly co-registered to the first image using Elastix 1.0.0.2 [20].

The ME model is defined as $S\left(t\right)=A\cdot exp\left(\frac{-t}{T_{2,ME}}\right)+\eta ,$ where A is the complex-valued signal amplitude, $T_{2,ME}$ denotes the average relaxation time, and η is the complex-valued Gaussian noise. The bi-exponential model (BE) assumes the existence of two pools of water in the same voxel, one pool with a fast relaxation time and the other with a slower relaxation time, and a parameter controlling the fraction of the total volume corresponding to the fast and slow components. The BE model is defined as $S\left(t\right)=A\cdot \left(f\cdot exp\left(\frac{-t}{T_{2,s}}\right)+\left(1-f\right)\cdot exp\left(\frac{-t}{T_{2,l}}\right)\right)+\eta$, where f denotes the fraction of the short relaxation time, $T_{2,s}$ denotes the short relaxation time, and $T_{2,l}$ the long relaxation time. In this model, the fast-relaxing pool is associated with macromolecule-bound water, and the slow-relaxing pool is associated with bulk water [13]. A previous study suggested that, in early OA, both the signal intensity and relaxation times of each pool may change differently, and these changes could potentially improve sensitivity [21].

**Figure 1:**
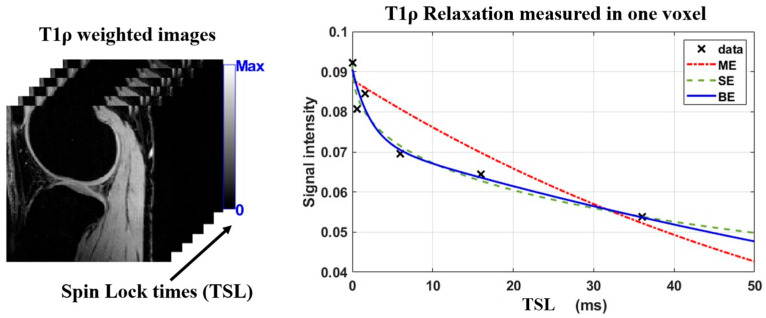
Exponential models used to fit the T_2_ data. Mono-exponential model shows worse agreement to the fitted data points from healthy volunteers than the more complex bi- and stretched-exponential models.

An alternative to the bi-exponential model is the stretched-exponential model (SE) [22], that does not assume a specific number of pools but rather models the heterogeneity of the voxel with one additional parameter compared to the ME model. It is defined as $S\left(t\right)=A\cdot exp\left(-{\left(\frac{t}{T_{2,SE}}\right)}^{\alpha }\right)+\eta$, α denotes the heterogeneity of voxel relaxation times. Its primary limitation is the difficulty in interpreting the heterogeneity parameter. Figure 1 illustrates the exponential models used here.

**Figure 2:**
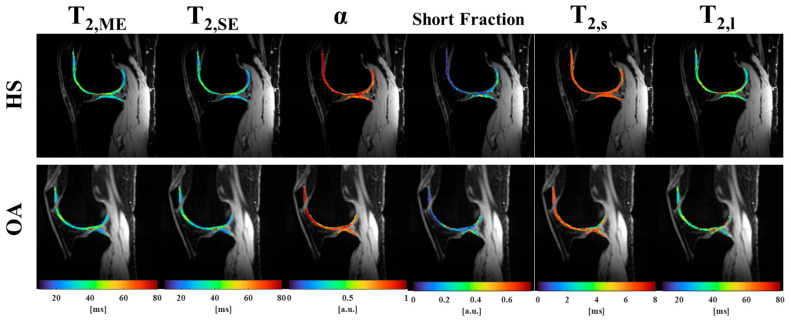
Representative T2 parameter maps from healthy and OA subjects.

Using a non-linear least squares method implemented through in-house developed scripts in MATLAB (The MathWorks Inc., Natick, MA, USA, R2024a), the complex-valued images were fitted to three exponential models: ME, SE, and BE models. From previous work [13,16], the model parameters were constrained to the following: $T_{2,ME}$ and $T_{2,SE}$ from 0.1 ms to 400 ms, *α* from 0.1 to 1.0, f from 0.01 to 0.99, BE $T_{2,s}$ from 0.1 to 10 ms, and BE $T_{2,l}$ from 20 to 300 ms. Figure 2 illustrates the obtained T_2_ maps of a healthy and an OA subjects.

**Figure 3:**
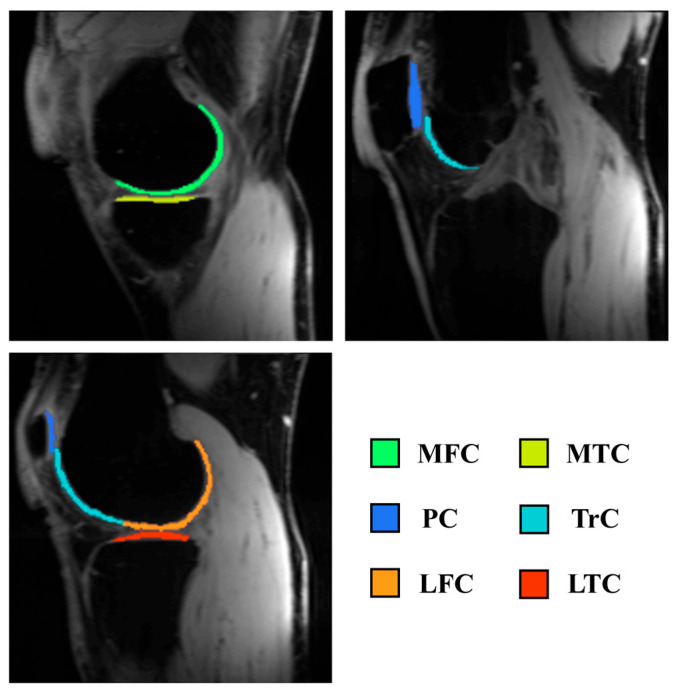
Example of cartilage segmentation labels based on the QIBA recommendations.

The cartilage was segmented using the Deep Open-Source Medical Image Analysis (DOSMA) framework segmentation tool [23] into three compartments: patellar cartilage, femoral cartilage, and tibial cartilage. Based on the Quantitative Biomarkers Alliance (QIBA) profile [24], the compartments were further divided into six regions: patellar cartilage (PC), trochlear cartilage (TrC), lateral and medial femoral cartilage (LFC and MFC), and lateral and medial tibial cartilage (LTC and MTC), as illustrated in Figure 3. The measurements were taken as the mean values of each cartilage segmented area and the mean values over the entire cartilage as a global area. The number of regions was selected to balance anatomical specificity with reproducibility and SNR constraints at the acquired spatial resolution, consistent with QIBA recommendations.

### 2.4. Statistical Analysis

The study was powered for the hypothesis of two-tailed group differences in globally averaged ME T_2_ with a Type I error rate of 5% and statistical power of 80%. Based on previous studies [10] and recommendations from the QIBA profile [24], a mean T_2_ of about 34 ms is expected for the HS group, with a standard deviation of 2.6 ms, and a minimum detectable effect of 7%, resulting in a sample size of 20 subjects per group.

To isolate disease-related changes from confounders, the quantitative parameters were adjusted for age using a linear regression model (Y ~ Age + Group + Age × Group). Given the limited sample size, additional stratified or interaction-based analyses (e.g., age × sex) were not feasible and are acknowledged as limitations. The nested cross-validation framework mitigates systematic bias by ensuring all adjustments are performed within training folds and preventing data leakage. Biological covariates, Sex and BMI, were not evaluated as potential confounders based on previous results [17].

For the univariate analysis, the Mann–Whitney U test was used to evaluate differences between groups for individual parameters. Effect sizes were calculated using Cliff’s delta. The multivariate analysis was performed for all three models to assess the diagnostic value of combining spatial and, for the SE and BE models, multi-parametric information. Linear Discriminant Analysis (LDA) was used to project the features into a single discriminant score [25]. Inside each fold, the average relaxation model parameters for the Global ROI were considered as inputs for the LDA, meaning the ME model was just rescaled, while the SE and BE models had 2 and 3 input features, respectively. For the Multi-ROI approach, the average relaxation model parameters for each ROI were considered as features for the LDA. For the ME model, this simply meant that the LDA received a feature vector of size 6, while for the BE model, the feature vector had a size of 18. These features span a multi-dimensional space that the LDA uses to separate the two groups. A regularized LDA [26,27] was employed to stabilize the covariance matrix estimation given the high feature-to-sample ratio and probable correlation between features. LDA weights for each model were recorded from each cross-fold iteration to assess stability. For a given weight distribution, a higher absolute mean value indicates an important feature, while a low variance indicates a stable estimation with good confidence.

Regularization and nested cross-validation were used to reduce overfitting risk of the model. This framework provides an unbiased estimate of model performance and is appropriate for exploratory, proof-of-concept studies.

Receiver Operating Characteristics (ROC) analysis [28] was performed using the data in the discriminator space, and performance was assessed by the AUC. Bootstrapping was performed to evaluate the 95% confidence interval (CI) for the AUC scores of the final model. To assess whether the models’ obtained AUC is better than random chance, i.e., H_0_:AUC = 0.5 and H_1_:AUC > 0.5, a permutation test was performed within the nested cross-validation framework. As this study focuses on early OA, modest AUC values are expected and do not reflect clinical diagnostic performance. Instead, the goal is to evaluate the relative sensitivity of BE or SE T2 versus ME in detecting early compositional changes.

For the test statistics on the LDA-combined data, a non-parametric MANOVA using the eigenvalues from the combination was used. Effect sizes were calculated using Cliff’s delta with the U statistic. DeLong’s test was used to evaluate pairwise differences in performance between models.

Calibration curves and Decision Curve Analysis were performed to assess the models’ clinical utility, with Brier Scores (BSs) used to compare the calibration curves. Feature stability was evaluated, and its significance was assessed by a permutation-based test.

The significance level was defined as *p* < 0.05, and the estimated *p*-values were corrected using false discovery rate (FDR) using the Benjamini–Hochberg method. All tests were performed in MATLAB (The MathWorks Inc., R2024a) environment using built-in methods and the Fathom toolbox [29].

## 3. Results

Table 2 summarizes the median and interquartile ranges for the three models’ parameters, along with *p*-values for the difference between the medians of the two groups and effect sizes.

**Figure 4:**
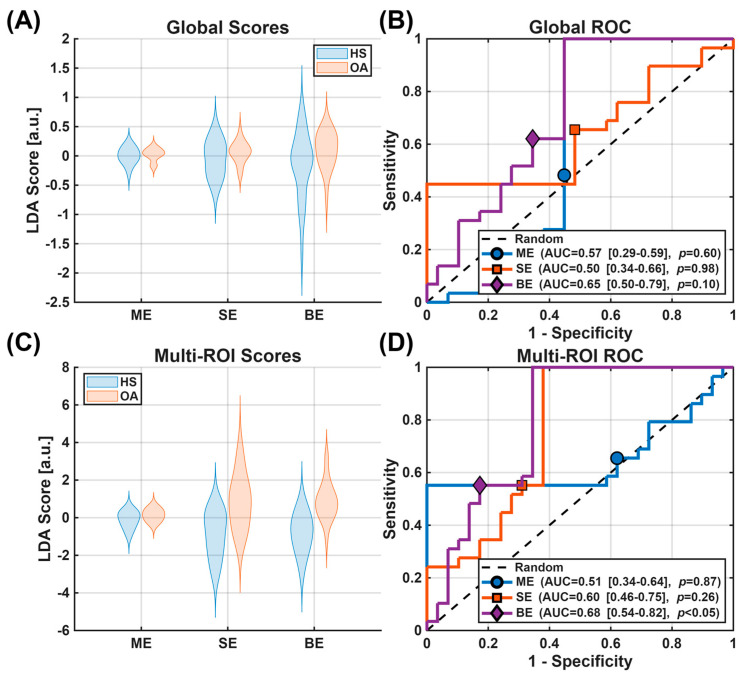
Group separation (**A**,**C**) and ROC analysis of the different models (**B**,**D**). When analyzing LDA for the Global ROI, group separation (**A**) was low for all models and the AUC for the ROC analysis (**B**) showed poor discriminative performance. For the Multi-ROI analysis, the group separation (**C**) for the SE and BE models, but in the ROC analysis (**D**), only the BE model achieved significant improvement over random chance, with a mean AUC of 0.68. Only the Multi-ROI BE model showed significant differentiation between groups.

Figure 4 illustrates the early OA detection performance of the LDA-combined biomarkers assessed with ROC analysis. The LDA-combined parameters on the global ROI resulted in mean AUCs and CIs of 0.57 [0.28, 0.59] (*p* = 0.60), 0.50 [0.35, 0.66] (*p* = 0.98), and 0.65 [0.50, 0.79] (*p* = 0.10) for the ME, SE, and BE models, respectively. When combining the model parameters over all sub-regions, the mean AUCs and CIs obtained were 0.51 [0.34, 0.64] (*p* = 0.87), 0.60 [0.46, 0.75] (*p* = 0.26), and 0.68 [0.54, 0.82] (*p* < 0.05), for the ME, SE, and BE models, respectively. DeLong’s tests showed no significant differences between models when considering the Global scores or the Multi-ROI scores. These AUC values are consistent with expectations for early OA, where subtle compositional changes limit discriminative performance, and should be interpreted within the exploratory, proof-of-concept scope of this study.

**Figure 5:**
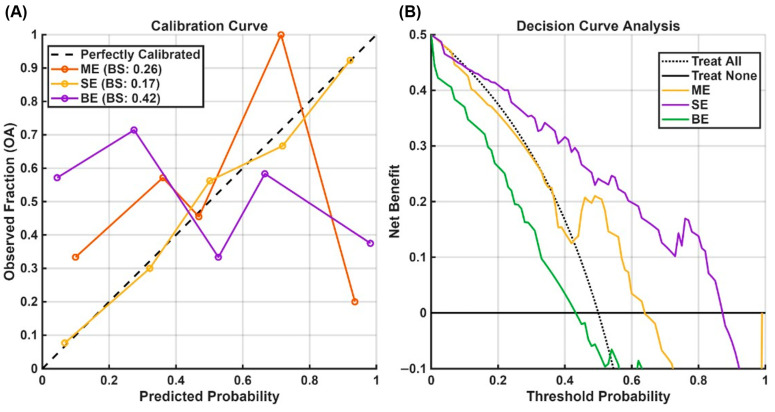
Calibration Curve (**A**) and Decision Curve Analysis (**B**). The models performed poorly on the calibration curve with the BE model presenting a Brier score result of 0.25, which means the best model is still inaccurate.

Figure 5 illustrates the performance of the multi-ROI models using calibration curves and decision curve analysis; the Global ROI models were not analyzed further. Although not intended to imply clinical readiness, the decision curve analysis provides insight into relative model behavior across threshold probabilities. The Brier Scores were 0.28, 0.29, and 0.25 for the ME, SE, and BE models, respectively. The decision curves indicate the net benefit of each model compared with standard “Treat All” (predicting all subjects as OA) and “Treat None” (predicting all subjects as healthy) strategies. The BE model demonstrated a modest net benefit within a threshold probability range of 0.3–0.7, where it lies above both standard strategies, whereas the SE model provided improved benefit over the Treat All and Treat None strategies between 0.4 and 0.6. In contrast, the ME model offered no advantage relative to these baseline strategies.

**Figure 6:**
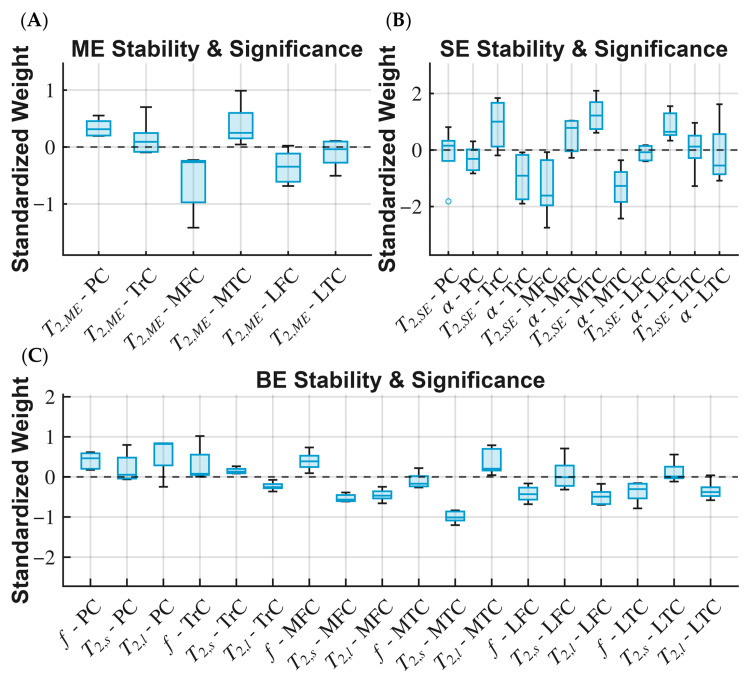
LDA Model weight distribution and stability as assessed across different cross-fold iterations. (**A**) Distribution of LDA feature weights for the ME model, where PC, MFC, and MTC showed weight distributions that did not change signs. (**B**) Distribution of weights for the SE model, where only the MTC features and α-LFC showed distributions with good stability. (**C**) The weight distributions for the BE model showed low variance for many of the features.

Figure 6 illustrates the distribution of LDA weights across the multi-ROI models, highlighting those that demonstrate importance and stability, as indicated by consistently high absolute values and low variance. Some variability in regional contributions was observed, which is expected given the biological heterogeneity of early OA and the modest sample size.

## 4. Discussion

The primary finding of this study is that multi-regional analysis using multi-exponential models shows improved sensitivity to early OA-related changes compared with the conventional ME model. This approach enhances OA detection in its early stages [30], before morphological changes such as cartilage loss are visible. This enhancement in early detection provides a window of opportunity for treatments that can slow or prevent structural deterioration. Although the ME model is widely used, our results indicate that multi-regional analyses of the BE and SE models provide improved discriminative performance relative to the ME approach. The novelty of this study lies in showing that, while T_2_ and T_1ρ_ relaxation times often exhibit similar mono-exponential behavior, the diagnostic performance of non-mono-exponential models differs between these two contrast mechanisms: the BE model provides superior performance for T_2_, whereas the SE model is more effective for T_1ρ_, as seen in [14]. The weight stability analysis suggests that certain regions contribute more to the discriminant signal, although variability remains expected given the modest sample size. The medial compartments (MFC and MTC) were consistently among the most important features in the models.

While statistically significant, the net benefit of the BE model is modest, which indicates the need for further refinement and validation of the technique before clinical translation.

A previous study [17] showed how the multi-ROI SE model improved detection of early OA and showed good calibration and net benefit curves when used with T_1ρ_ data, while the BE model failed to show any clinical benefit. The results presented here confirm that globally averaging T_2_ obscures the local signal changes associated with early degeneration, which aligns with the known compartmental distribution of knee OA [31]. But they also demonstrate that the SE model does not perform well for T_2_, and conversely, the BE model performs better for T_2_ than for T_1ρ_.

These findings further support the view that spatially specific cartilage assessment may offer advantages over global averaging when evaluating early OA [7,32,33]. By considering different compartments as distinct variables, patterns of disease that could be better predictors than the magnitude of a global average can be found.

Beyond diagnostic performance, improved sensitivity to subtle, region-specific cartilage changes may have practical clinical value. Earlier identification of focal abnormalities could help guide conservative management decisions and support more precise patient selection for disease-modifying OA trials. From a surgical perspective, better characterization of compartment-specific degeneration may assist in refining indications for joint-preserving procedures and in monitoring postoperative recovery.

There was a sex imbalance in the OA group. Although sex was not included as a covariate in the final models due to limited statistical power and prior evidence suggesting minimal impact on T values, this imbalance remains a limitation.

This study used a small sample size (*N* = 52), which limited the statistical power and generalization of the results presented. Given the significant differences in demographics, this small sample size could lead to residual confounding effects in the results, reducing the capacity to detect subtle differences. A follow-up study with a larger sample size is required to further validate the results presented here, preferably with a more evenly distributed sample. Still, this work served as proof-of-concept for the combination of multi-parametric and sub-region analysis for the early detection of OA and further shows how the different models perform differently for different contrast mechanisms. T_2_ is more susceptible to the magic angle effect due to the dipolar interactions of fiber orientation with respect to B_0_ [21], compared to T_1_ and, to a lesser extent, T_1ρ_; therefore, another limitation of our study is in accounting for differences in orientation of cartilage in the knee.

Variability in model performance likely reflects the biological heterogeneity of early OA, where structural and compositional changes progress non-uniformly across individuals. Subgroup analyses were not feasible due to sample size and are acknowledged as an area for future work.

This study was conducted at a single center using a single MRI scanner and vendor. Because quantitative MRI measurements can vary with scanner vendor and magnetic field strength, external validation is needed to confirm the generalizability of these findings. Future multi-center studies will be important to assess reproducibility across vendors and imaging platforms.

## 5. Conclusions

This study demonstrated that sub-regional analysis using bi-exponential T_2_ mapping significantly enhances the detection of early osteoarthritis compared with the conventional mono-exponential approach. In contrast, the stretched-exponential model showed limited effectiveness. These results further support the use of multi-regional, multi-exponential models as potential imaging biomarkers for early OA pending validation in larger cohorts, and underscore the distinct performance differences between T_2_ and T_1ρ_ modeling.

## Figures and Tables

**Table 1:** Demographics of the recruited subjects. Significant differences in the sex and age distributions were found, but no difference in body mass index (BMI).

Group	Total Recruited	Females	Age	BMI
Healthy Subjects	26	10	51.5	27.3
Osteoarthritic patients	26	19	61.8	27.8
***p*-value**	**-**	<**0.05**	<**0.05**	**0.92**

**Table 2:** Median parameter values (ms) and interquartile ranges (ms) for all three models, mono-, stretched-, and bi-exponential (ME, SE, and BE), for both healthy subject (HS) (gray rows) and early osteoarthritis (OA) (white rows) groups, along with the *p*-values from the Mann–Whitney U-test between groups and effect size (es). Bold values indicate significant differences. The represented regions of interest are: medial femoral cartilage (MFC), medial tibial cartilage (MTC), patellar cartilage (PC), trochlear cartilage (TrC), lateral femoral cartilage (LFC), and lateral tibial cartilage (LTC).

	Global	PC	TrC	MFC	MTC	LFC	LTC
ME T_2_	42.4 [25.4–58.9]	8.5 [0.4–28.1]	34.3 [12.4–53.0]	23.8 [20.8–51.0]	55.6 [48.6–74.2]	53.5 [32.0–75.7]	59.4 [40.0–70.5]
	39.2 [29.4–51.6]	14.5 [5.7–19.5]	35.9 [19.5–58.5]	25.4 [20.2–59.5]	66.2 [45.8–81.3]	50.3 [34.1–67.6]	59.4 [38.2–81.8]
	*p* = 0.97	*p* = 0.69	*p* = 0.74	*p* = 0.69	*p* = 0.88	*p* = 0.99	*p* = 0.97
	d = 0.01	d = 0.16	d = 0.12	d = 0.16	d = 0.04	d = 0.00	d = 0.01
SE T_2_	24.7 [12.3–34.1]	4.7 [0.2–14.1]	17.8 [9.0–32.3]	24.9 [12.4–47.4]	33.5 [24.4–43.3]	35.5 [18.7–48.6]	34.4 [19.9–45.7]
	24.7 [18.9–33.9]	6.3 [2.3–11.2]	21.6 [14.0–34.6]	20.8 [11.2–39.8]	38.2 [28.4–57.0]	32.6 [23.0–44.3]	38.7 [23.7–51.7]
	*p* = 0.88	*p* = 0.79	*p* = 0.56	*p* = 0.75	*p* = 0.56	*p* = 0.97	*p* = 0.74
	d = 0.06	d = 0.09	d = 0.21	d = 0.11	d = 0.22	d = 0.02	d = 0.12
SE α	0.31 [0.19–0.51]	0.07 [0.01–0.33]	0.29 [0.09–0.46]	0.41 [0.21–0.59]	0.67 [0.47–0.76]	0.34 [0.21–0.46]	0.56 [0.45–0.69]
	0.30 [0.23–0.38]	0.06 [0.01–0.22]	0.25 [0.15–0.37]	0.29 [0.17–0.53]	0.51 [0.34–0.57]	0.36 [0.26–0.46]	0.51 [0.38–0.62]
	*p* = 0.77	*p* = 0.74	*p* = 0.88	*p* = 0.62	*p* = 0.11	*p* = 0.88	*p* = 0.41
	d = 0.10	d = 0.11	d = 0.04	d = 0.19	d = 0.41	d = 0.04	d = 0.27
BE Short Fraction	0.22 [0.17–0.3]	0.14 [0.09–0.2]	0.17 [0.14–0.2]	0.24 [0.18–0.3]	0.27 [0.22–0.3]	0.24 [0.21–0.4]	0.26 [0.21–0.3]
	0.21 [0.19–0.29]	0.16 [0.13–0.21]	0.19 [0.17–0.24]	0.26 [0.20–0.31]	0.25 [0.19–0.31]	0.26 [0.18–0.30]	0.20 [0.16–0.30]
	*p* = 0.89	*p* = 0.56	*p* = 0.62	*p* = 0.69	*p* = 0.77	*p* = 0.56	*p* = 0.41
	d = 0.04	d = 0.23	d = 0.19	d = 0.15	d = 0.10	d = 0.21	d = 0.27
BE Short T_2_	5.7 [5.1–6.0]	6.0 [5.5–6.3]	5.9 [5.2–6.1]	5.7 [5.1–6.0]	5.6 [5.0–6.0]	5.4 [4.7–6.0]	5.7 [5.0–6.1]
	5.4 [5.1–5.7]	5.8 [5.6–6.2]	5.6 [5.3–6.1]	5.2 [4.6–5.7]	5.4 [4.8–5.6]	5.2 [4.8–5.5]	5.4 [5.0–5.8]
	*p* = 0.56	*p* = 0.74	*p* = 0.65	*p* = 0.18	*p* = 0.23	*p* = 0.79	*p* = 0.69
	d = 0.21	d = 0.12	d = 0.18	d = 0.35	d = 0.33	d = 0.08	d = 0.14
BE Long T_2_	54.1 [50.5–77.7]	58.1 [48.9–80.0]	58.5 [47.1–84.4]	55.3 [43.9–85.5]	49.4 [42.1–53.3]	65.4 [51.6–91.8]	47.3 [42.2–54.5]
	54.8 [49.5–67.5]	55.0 [46.6–66.6]	56.0 [47.0–73.5]	57.0 [44.5–76.4]	55.2 [47.2–74.0]	47.2 [38.2–61.1]	39.8 [30.9–47.3]
	*p* = 0.69	*p* = 0.69	*p* = 0.88	*p* = 0.88	*p* = 0.18	*p* = 0.07	*p* = 0.11
	d = 0.14	d = 0.15	d = 0.05	d = 0.05	d = 0.36	d = 0.48	d = 0.41

## Data Availability

The data supporting the findings of this study are available from the corresponding author upon reasonable request. The data are not publicly available due to privacy and ethical considerations.

## Abbreviations

The following abbreviations are used in this manuscript:

AICc	Corrected Akaike Information Criterion
AUC	Area Under the Receiver Operating Characteristic Curve
BE	Bi-exponential
BMI	Body Mass Index
BS	Brier Scores
CIs	Confidence Intervals
CRLB	Cramer–Rao Lower Bound
DOSMA	Deep Open-Source Medical Image Analysis
ES	Effect Size
FDR	False Discovery Rate
FNIH	Foundation for the National Institutes of Health
HIPAA	Health Insurance Portability and Accountability Act
HS	Healthy Subject (also referred to as Healthy Volunteer)
IRB	Institutional Review Board
KL	Kellgren–Lawrence
LDA	Linear Discriminant Analysis
LFC	Lateral Femoral Cartilage
LTC	Lateral Tibial Cartilage
MANOVA	Multivariate Analysis of Variance
ME	Mono-exponential
MFC	Medial Femoral Cartilage
MRI	Magnetic Resonance Imaging
MTC	Medial Tibial Cartilage
OA	Osteoarthritis
PC	Patellar Cartilage
PROMs	Patient-Reported Outcome Measures
QIBA	Quantitative Biomarkers Alliance
ROC	Receiver Operating Characteristic
ROI	Region Of Interest
SE	Stretched-exponential
SENSE	Soft-sensitivity Encoding
T_1ρ_	Spin-lattice relaxation time in the rotating frame
T_2_	Spin-spin relaxation time
TrC	Trochlear Cartilage
